# Isolated Cardiac Sarcoidosis Presenting as High-Degree Atrioventricular Block

**DOI:** 10.7759/cureus.62685

**Published:** 2024-06-19

**Authors:** Ryian Owusu, Hazem Alakhras, Kateryna Strubchevska, Daniel G Walsh

**Affiliations:** 1 Internal Medicine, Oakland University William Beaumont School of Medicine, Rochester, USA; 2 Internal Medicine, Corewell Health William Beaumont University Hospital, Royal Oak, USA; 3 Cardiovascular Medicine, Corewell Health William Beaumont University Hospital, Royal Oak, USA

**Keywords:** atrioventricular block, cardiac positron emission tomography, cardiac magnetic resonance, bradycardia, isolated cardiac sarcoidosis

## Abstract

Isolated cardiac sarcoidosis is a rare phenomenon of systemic sarcoidosis, with presentation ranging from asymptomatic to sudden cardiac death. Controversy exists on diagnostic and therapeutic options, creating an ongoing challenge for clinicians in providing patient care. A 79-year-old male presented status post looposcopy and interval ureteral stent replacement with sinus bradycardia and high-degree atrioventricular block. A comprehensive examination was performed that conclusively ruled out common etiologies of atrioventricular block, including coronary artery disease, electrolyte abnormalities, and medications. This prompted an investigation using advanced cardiac imaging modalities that demonstrated cardiac sarcoidosis. Computed tomography of the chest was negative for lymphadenopathy or infiltrates indicative of pulmonary involvement. The lack of extracardiac manifestations, in combination with imaging findings, led to a probable diagnosis of isolated cardiac sarcoidosis. The patient underwent biventricular implantable cardioverter defibrillator placement and was started on oral corticosteroids.

## Introduction

Sarcoidosis is an inflammatory disorder of unknown etiology characterized by the presence of non-caseating granulomas in various organ systems. Clinical manifestations frequently develop in African American females, between the ages of 20-60, with peaks in a bimodal distribution [1]. Although the exact mechanism is unknown, it is postulated that genetically predisposed individuals develop disease symptomatology following exposure to an environmental trigger [2].

At presentation, an estimated 90-95% of patients will have pulmonary involvement with associated chest imaging findings of bilateral hilar lymphadenopathy [1]. However, cardiac sarcoidosis is less prevalent affecting 5-8% of patients with systemic sarcoidosis and appearing in isolation in approximately 25% of cardiac sarcoidosis cases [3]. Cardiac sarcoidosis is highly variable in presentation, with asymptomatic cases, conduction diseases, arrhythmias, heart failures, and sudden cardiac deaths all being reported [4].

An atrioventricular block is a conduction disease that is characterized by an interruption of transmission from the atria to the ventricles [5]. This disturbance may be intermittent or permanent and is further classified into the following: first, second (Mobitz type I and Mobitz type II), and third-degree atrioventricular block [5]. Etiologies are varied including idiopathic, ischemic heart disease, congenital heart disease, electrolyte abnormalities, medications, cardiac procedures, or infiltrative processes such as sarcoidosis [5]. The most common are idiopathic fibrosis and sclerosis, accounting for 50% of cases, and coronary artery disease, accounting for 40% [5].

The diagnosis of cardiac sarcoidosis is particularly challenging due to nonspecific symptoms and diagnostic criteria with inadequate sensitivity [3]. Additionally, the management of cardiac sarcoidosis is complex and requires a multidisciplinary approach. Corticosteroids have been the mainstay of treatment, although, in cardiac sarcoidosis, management further aims to correct complications secondary to the disease process [6]. This incorporates a multitude of therapies such as immunosuppressants, heart failure guideline-recommended interventions, implantable cardioverter-defibrillators, and permanent pacemakers [4].

## Case presentation

A 79-year-old Caucasian male, with a medical history significant for coronary artery disease, hypertension, hyperlipidemia, bladder and prostate cancer status post cystoprostatectomy, and ureteral-ileal loop anastomotic stricture, presented for looposcopy and interval ureteral stent replacement for chronic hydronephrosis. Following an uncomplicated procedure, the patient was transitioned to the post-anesthesia care unit where he was deemed hemodynamically stable and producing appropriate urine through an urostomy bag. On telemetry, the patient was noted to have intermittent pauses and ectopic beats. Further, his heart rate decreased from 60s to 40s and remained in that range. The patient was asymptomatic with other vital signs within normal limits. He denied symptoms of chest pain, lightheadedness, dizziness, or syncope. The patient had no history of medications that predisposed to bradycardia, such as beta-blockers or calcium channel blockers.

On examination, he was alert, oriented, and in no acute distress. A cardiac exam was significant for bradycardia with no additional heart sounds or murmurs. There was normal jugular venous pressure. Pedal pulses were palpable bilaterally, and the chest was clear to auscultation.

Labs and imaging

**Figure 1:**
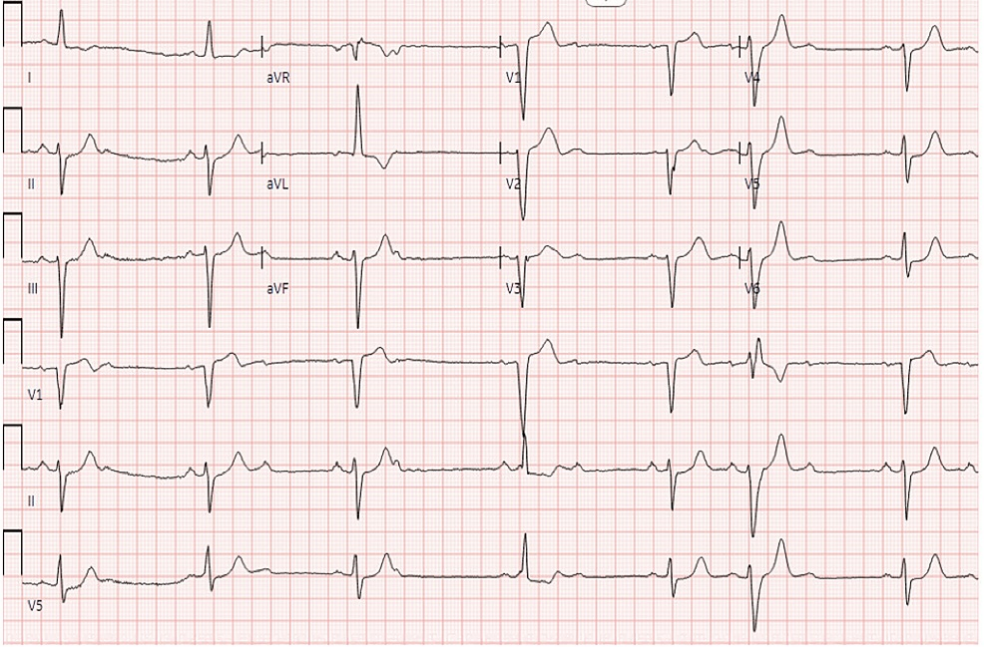
Electrocardiogram Normal sinus rhythm with a heart rate of 41 beats per minute, second-degree atrioventricular block (Mobitz II), blocked premature atrial complexes, and left bundle branch block

**Figure 2:**
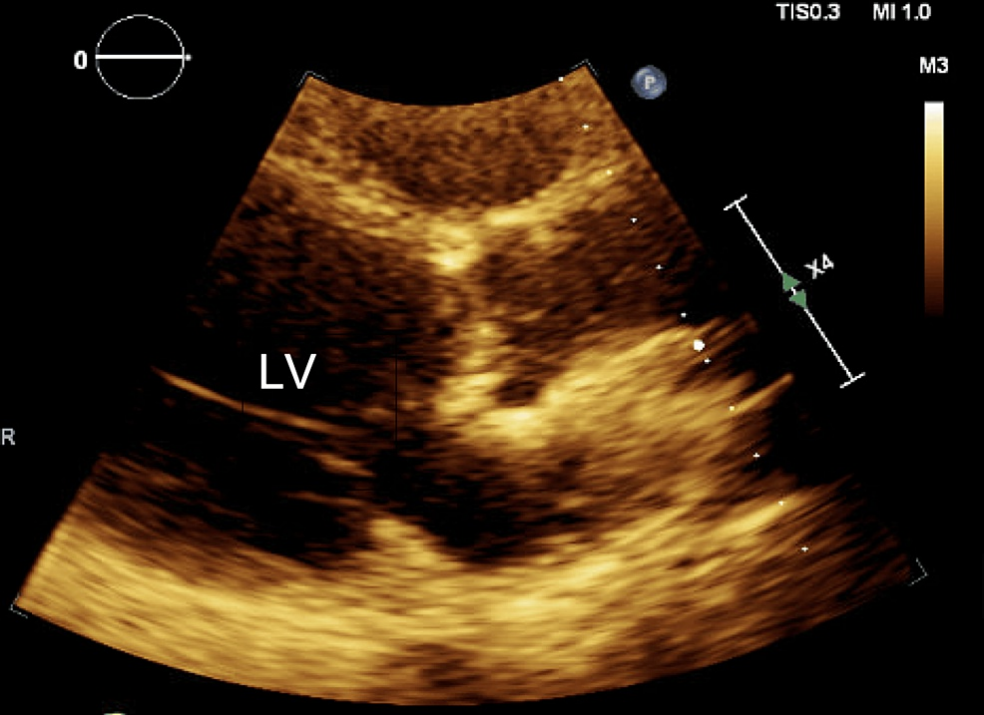
Transthoracic echocardiogram Transthoracic echocardiogram showing left ventricular (LV) enlargement

**Figure 3:**
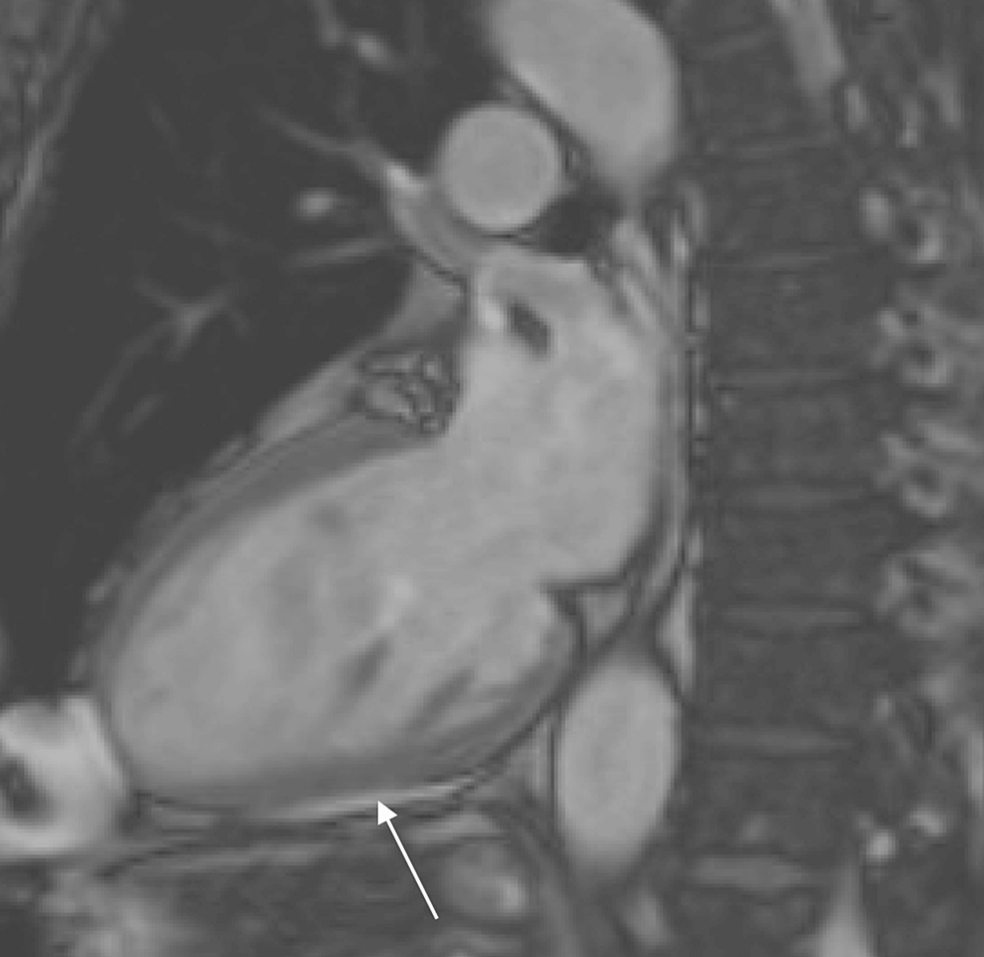
Cardiac magnetic resonance Cardiac magnetic resonance showing delayed gadolinium enhancement (arrow) of the inferior myocardium

**Figure 4:**
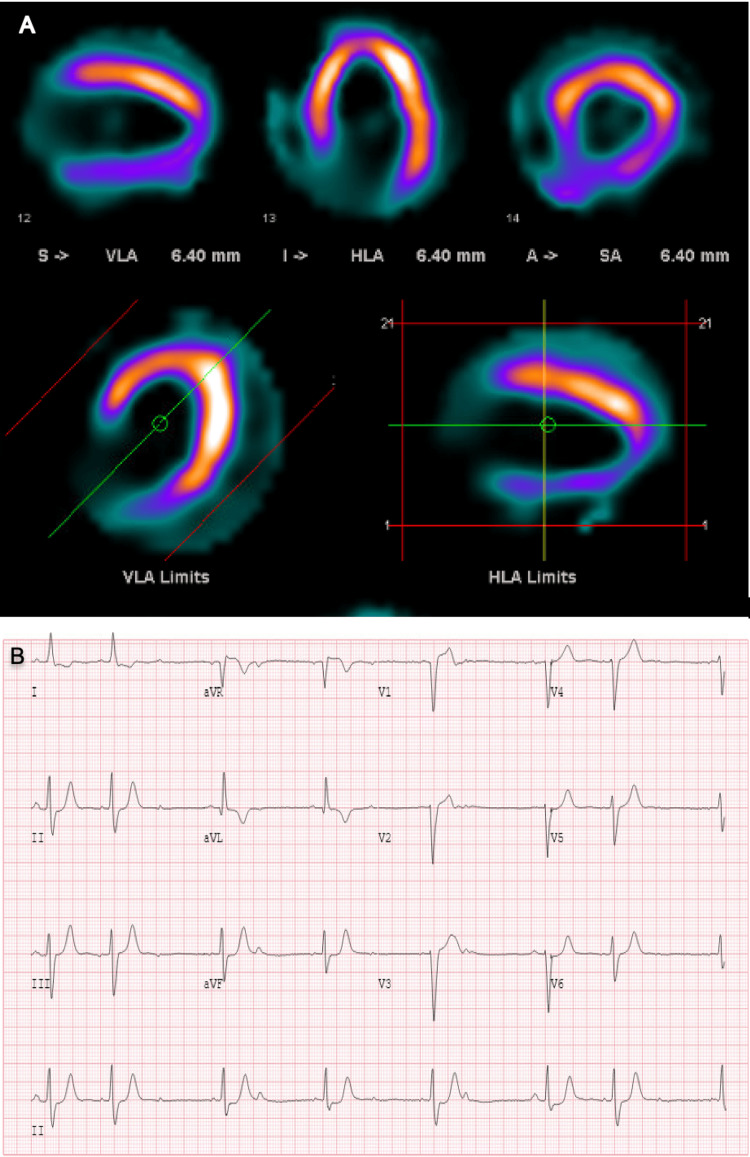
Pharmacologic stress myocardial perfusion imaging (A) Reduced count density in the inferior and inferior-apical regions of the left ventricle. (B) Electrocardiogram showing a left bundle branch block and second-degree heart block during infusion

**Figure 5:**
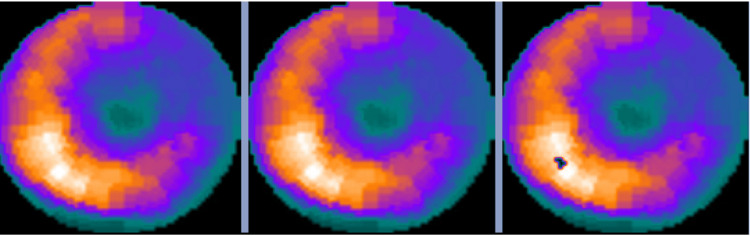
Myocardial positron emission tomography Myocardial positron emission tomography showing metabolic uptake in the left ventricular myocardium

The initial complete blood count, comprehensive metabolic panel, and troponin levels were unremarkable. Electrocardiogram demonstrated normal sinus bradycardia (heart rate of 41 beats per minute) with non-conducted premature atrial contractions, Mobitz II atrioventricular block with a 2:1 conduction, and left bundle branch block alternating with right bundle branch block (Figure 1). The echocardiogram showed global hypokinesis, left ventricle enlargement (left ventricular internal dimension at end-diastole of 6.7 cm), and left ventricular ejection fraction of 35% (Figure 2). These findings indicated left ventricular dysfunction, prompting additional imaging to determine the underlying cause. Cardiac magnetic resonance imaging showed delayed gadolinium enhancement of the inferior and inferior-lateral myocardium (Figure 3). This is a nonspecific finding that could be attributed to inflammatory (i.e., myocarditis) or infiltrative (i.e., sarcoidosis) processes. Pharmacologic stress myocardial perfusion imaging demonstrated reduced density in the inferior and inferior-apical area of the left ventricle, as well as global hypokinesis with left ventricular ejection fraction of approximately 38% (Figure 4A). Additionally, the electrocardiogram showed rate-related left bundle branch block and second-degree heart block before and during infusion (Figure 4B). The diminished density indicates a reduction in blood flow to associated areas of the heart during stress with accompanying conduction abnormalities. Myocardial positron emission tomography (N-13-ammonia/18F-fluorodeoxyglucose) showed metabolic uptake in the left ventricular myocardium, suspicious for inflammation, or sarcoidosis involvement, with no extracardiac sarcoidosis identified (Figure 5). Cardiac catheterization revealed non-obstructive coronary artery disease, while computed tomography of the chest was unremarkable for any hilar lymphadenopathy.

Management

During hospitalization, the patient was continued on home medications of aspirin and rosuvastatin. Although a definitive diagnosis with histological evidence was not obtained, the patient’s clinical and supporting imaging findings were highly suspicious for isolated cardiac sarcoidosis leading to the initiation of intravenous steroids. Therapy was transitioned to daily oral prednisone and trimethoprim/sulfamethoxazole for *Pneumocystis jirovecii* prophylaxis. The patient later underwent biventricular implantable cardioverter-defibrillator placement, given his findings of high-grade atrioventricular block. He subsequently was started on sacubitril/valsartan and carvedilol.

## Discussion

Isolated cardiac sarcoidosis remains a diagnostic challenge due to its highly diverse clinical manifestations and rarity in patient presentation. Therefore, healthcare providers must have a high index of suspicion as undiagnosed cardiac sarcoidosis may be fatal. There are currently no gold standard diagnostic guidelines for establishing cardiac sarcoidosis. However, proposed algorithms have been created as a framework for working up and reaching this diagnosis.

The Japanese Circulation Society developed specific guidelines detailing diagnostic criteria for isolated cardiac sarcoidosis [7]. First, the prerequisite of absent clinical or imaging findings indicative of sarcoidosis in extracardiac organs must be fulfilled [7]. This includes a detailed examination of commonly affected organ systems (i.e., respiratory, skin, eyes), followed by imaging with chest computed tomography, ^18^F-fluorodeoxyglucose positron emission tomography, and/or 67Ga scintigraphy, confirming the absence of internal involvement [7]. After completion of prerequisites, patients are either diagnosed via histology or clinical findings [7]. Histological confirmation of non-caseating granulomas is the most definitive means of diagnosis, albeit risks and benefits must be weighed for endomyocardial biopsy due to the low sensitivity of 20-30% and potential procedural complications [6]. Given this drawback, patients may be diagnosed through clinical findings found in imaging studies, such as electrocardiogram, echocardiogram, cardiovascular magnetic resonance, ^18^F-fluorodeoxyglucose positron emission tomography, and/or myocardial perfusion scintigraphy [7]. The Japanese Circulation Society created major and minor criteria, where at least two or more of the five major criteria or one of the major and two or more of the three minor criteria must be satisfied [7]. In regard to our patient, he met four out of five major criteria (high-grade atrioventricular block, left ventricular ejection fraction less than 50%, abnormal tracer accumulation on ^18^F-fluorodeoxyglucose positron emission tomography, and delay contrast enhancement on magnetic resonance imaging) and two out of three minor criteria (abnormal electrocardiogram and perfusion defects on myocardial perfusion scintigraphy). Therefore, a diagnosis of isolated cardiac sarcoidosis was appropriately reached, supported by highly suggestive clinical and imaging findings and absent extracardiac involvement.

The diagnostic dilemma of cardiac sarcoidosis stems from the patchy involvement of the myocardium and the lack of highly sensitive and specific noninvasive testing modalities [8]. Advanced imaging studies, such as gadolinium-enhanced magnetic resonance imaging and positron emission tomography, are becoming increasingly popular; however, limitations of high cost, limited availability, and the need for expert interpretation must be considered [8]. Late gadolinium enhancement on cardiac magnetic resonance is well associated with myocardial fibrosis, while cardiac positron emission tomography, available as myocardial perfusion or ^18^F-fluorodeoxyglucose radiotracer, is correlated with the acute inflammatory phase of cardiac sarcoidosis [3]. In other words, cardiac magnetic resonance has higher sensitivity during the chronic fibrotic stage and positron emission tomography early in the disease process during acute inflammation [3]. Considering the clinical variability and nonspecific symptoms, determining the onset of the disease becomes challenging. To mitigate this issue, the combined use of these two imaging studies creates a synergistic effect, where the weakness of one is the strength of the other [3]. This provides the highest degree of accuracy when identifying isolated cardiac sarcoidosis by clinical measures [3].

This case emphasizes the importance of conducting a thorough workup for patients with new onset arrhythmia. Our patient, who presented with a high-grade atrioventricular block, underwent a comprehensive diagnostic evaluation to determine the underlying cause. This included negative cardiac enzymes, cardiac catheterization, complete blood count, comprehensive metabolic panel, and medication review. Given the unremarkable laboratory tests and examinations, advanced cardiac imaging was performed. Finally, cardiac magnetic resonance and myocardial positron emission tomography revealed findings indicative of cardiac sarcoidosis, confirming our diagnosis. Idiopathic fibrosis stands as the most common etiology of atrioventricular block; however, it serves as a diagnosis of exclusion. It is imperative to eliminate common etiologies, as failure to do so can result in unfavorable patient outcomes. In our case, maintaining high clinical suspicion was critical in proceeding with advanced cardiac imaging, ultimately leading to the diagnosis. This facilitated the timely initiation of therapeutic modalities for our patient.

There is a lack of consensus on the management of cardiac sarcoidosis; however, corticosteroids have been at the forefront of medical therapy [6]. It is recommended that patients remain on low-dose corticosteroids for at least one year, but recurrence is common regardless of the initial resolution of symptoms [6]. Limited data exist on the efficacy of corticosteroids, as well as other immunosuppressant agents [6]. Implantable cardioverter-defibrillators are recommended as primary prevention of sudden cardiac death for patients with high-grade atrioventricular block, ventricular arrhythmias, low ejection fraction (<35%), and structural heart disease [6].

## Conclusions

Isolated cardiac sarcoidosis is a rare entity characterized by diverse and heterogeneous presentations. This case highlights the importance of isolated cardiac sarcoidosis as a differential diagnosis in patients presenting with high-degree atrioventricular block, following the exclusion of more common etiologies such as acute coronary syndrome, medications, and electrolyte disturbances. It is crucial for clinicians to adopt a low threshold for advanced cardiac imaging in patients exhibiting new arrhythmia of unknown etiology.
